# Supplementary material for: KLF4 Suppresses the Progression of Hepatocellular Carcinoma by Reducing Tumor ATP Synthesis through Targeting the Mir-206/RICTOR Axis
Source: Int J Mol Sci. 2024 Jun 28;25(13):7165. doi: 10.3390/ijms25137165 (PMC11240942; doi:10.3390/ijms25137165)
Supplement: Supplementary file 1 [file ijms-25-07165-s001.zip › ijms-3049418-supplementary.pdf]

Supplementary Table 1. Correlation of clinicopathological parameters and KLF4 expression in tumor in our cohort.

Characteristics	N n=130	KLF4 expression High n=70	Low n=60	*P*
Age in years				
≥60	35	19	16	0.951
＜60	95	51	44	
Sex				
Male	114	63	51	0.387
Female	16	7	9	
Tumor number				
Solitary	102	53	49	0.410
Multiple	28	17	11	
Tumor size in cm				
≥5	74	39	35	0.764
＜5	56	31	25	
Cirrhosis				
Yes	93	46	47	0.112
No	37	24	13	
MVI				
Yes	32	12	20	**0.033***
No	98	58	40	
ALB <35 g/L				
Yes	10	3	7	0.115
No	110	67	53	
ALT≥50 U/L				
Yes	34	17	17	0.601
No	96	53	43	
AST≥40 U/L				
Yes	46	21	25	0.165
No	84	49	35	
differentiation status				
Well/moderate differentiation	104	62	42	**0.008***
Poor differentiation	26	8	18	

ALB albumin, ALT alanine aminotransferase, AST aspartate transaminase. *$P < 0.05$.

Supplementary Table 2.Univariate Cox regression analyses for overall survival and recurrence-free survival in our cohort.

	Overall Survival		Recurrence-free survival	
	**HR (95% CI)**	***P***	**HR (95% CI)**	***P***
KLF4 expression	0.35 (0.20, 0.62)	**<0.001***	0.33 (0.19, 0.56)	**<0.001***
Age ≥ 60 years	1.09 (0.60, 1.99)	0.773	1.24 (0.72, 2.15)	0.442
Male	2.64 (0.82, 8.48)	0.102	1.49 (0.64, 3.46)	0.355
Multiple tumors	2.28 (1.28, 4.08)	0.005	1.6 (0.89, 2.86)	0.117
tumor diameter≥5 cm	1.33 (0.79, 2.31)	0.307	1.77 (1.04, 3.01)	0.034
cirrhosis	0.97 (0.53, 1.75)	0.907	0.66 (0.39, 1.12)	0.125
MVI	1.8 (1.01, 3.20)	0.047	1.37 (0.77, 2.42)	0.281
ALB <35 g/L	4.69 (2.18, 10.08)	**<0.001***	3.02 (1.37, 6.69)	**0.006***
ALT≥50 U/L	0.77 (0.40, 1.47)	0.429	0.88 (0.49, 1.58)	0.672
AST ≥40 U/L	1.03 (0.59, 1.81)	0.903	0.98 (0.58, 1.66)	0.942
TBIL≥17.1 umol/L	0.81 (0.42, 1.54)	0.52	1.01 (0.57, 1.79)	0.966
differentiation	1.8 (0.97, 3.32)	0.059	1.3 (0.69, 2.44)	0.419

ALB albumin, ALT alanine aminotransferase, AST aspartate transaminase, TBIL total bilirubin.

*P < 0.05.

Supplementary Table 3. The primers used for qPCR assay in present study

Primer names	Forward (5'-3')	Reverse (5'-3')
KLF4	CATCTCAAGGCACACCTGCGAA	TCGGTCGCATTTTTGGCACTGG
ACTB	CACCATTGGCAATGAGCGGTTC	AGGTCTTTGCGGATGTCCACGT
Raptor	GATCGTCAACAGCTATCACACGG	CGAGTCGAAGTTCTGCCAGATC
MLST8	CAGGTGAATGCCTTGGAGGTCA	TTGTTGACGCCGTCGTAGCTGA
MTOR	AGCATCGGATGCTTAGGAGTGG	CAGCCAGTCATCTTTGGAGACC
PRAS40	CGGTCATCAGATGAGGAGAATGG	GCTTCTGGAAGTCGCTGGTGTT
DEPTOR	ACTGGCTGGTTCAGGAAGGTGA	GCTGTCCACAAATGGGTGCTTG
RICTOR	GCCAAACAGCTCACGGTTGTAG	CCAGATGAAGCATTGAGCCACTG
Protor 1	CCTTCACCCATTCCTGCATCCT	GGTTCAGCAGAGGCGTGTTGTA
Sin1	CAGGACAGACTGCTGCCAATGA	GGCAGTAGGCACTGACATTGTC
mirR206-mo1	CTACAAGGTTGGGCTTGGCA	GCCAGGCTGAGCTTCATACAT
mirR206-mo2	CTCAGCCTGGCTCATGAATAG	TGTGAGGAGTGAGGGTTTGC
mirR206-mo3	TGAGCCATTGGATGATTCCCA	GTAACTCTGCGCTTGGGGT
mirR206-mo4	GTTTTTAACCACCCCAGCCAG	CTGGAGGGAGATGGGGTTTTG
mirR206-mo5	GGGGGCCAACTCTTCCTTTG	GGACATTGGCAGCTGTTGAG
